# The World Health Organization ageism towards older persons scale: preliminary validation of a novel measure of ageist stereotypes, prejudices, and discrimination in four different countries

**DOI:** 10.1093/ageing/afaf384

**Published:** 2026-01-24

**Authors:** Liat Ayalon, M Clara Maria P de Paula Couto, Klaus Rothermund, Jana Nikitin, Xuefei Li, Zhuoni Xiao, Aja Louise Murray

**Affiliations:** Louis and Gabi Weisfeld School of Social Work, Max and Anna Web, Ramat Gan, Israel 52900, Israel; Friedrich-Schiller-Universität Jena, Jena, Thuringia, Germany; Friedrich-Schiller-Universität Jena, Jena, Thuringia, Germany; University of Vienna, Vienna, Vienna, Austria; University of Edinburgh, Edinburgh, Scotland, United Kingdom of Great Britain and Northern Ireland; University of Edinburgh, Edinburgh, Scotland, United Kingdom of Great Britain and Northern Ireland; University of Edinburgh - Department of Psychology, Edinburgh, United Kingdom of Great Britain and Northern Ireland

**Keywords:** psychometrics, assessment, ageism, discrimination, prejudices, stereotypes, older people

## Abstract

This study presents the preliminary validation of the WHO Ageism Towards Older Persons Scale (WHO-A-TOPS), a new measure designed to comprehensively assess ageism, whilst capturing its three dimensions (e.g. stereotypes, prejudices, and discrimination based on age). The study evaluated the structural validity, measurement invariance, internal consistency, and construct validity of the WHO-A-TOPS. Data were collected from four countries: Czech Republic, Germany, Israel, and the United Kingdom, with a total sample of 1778 participants aged 20–90 years. Through an iterative process, a 10-item one-factor model was identified, demonstrating acceptable partial scalar measurement invariance across the four countries and invariance across different age groups. Hence, indicating that the new measure can capture a common construct across the four investigated countries and the three age groups. The final 10-item scale captures all three dimensions of ageism: stereotypes, prejudices, and discrimination. The new tool represents an exceptional attempt to develop a measure of high psychometric properties following current state-of-the-art guidelines. The tool can be used across different countries and age groups. The study discusses the implications of these findings for ageism research and practise, highlighting the importance of cross-country validation and the complexities of measuring ageism’s multifaceted nature.

## Key Points

- The research identified a 10-item, one-factor solution for the scale.
- The new measure captures a common construct across countries and age groups.
- The new measure is a reliable and valid tool for assessing ageism.

## Introduction

Ageism is a complex and pervasive phenomenon encompassing not only discriminatory behaviours but also the stereotypes and prejudices that shape how individuals perceive and treat others and oneself based on their age. Whilst its negative effects, especially on older persons, are well documented, existing measures are often unidimensional, overlooking the full scope of ageism. Relying on data from four different countries, we present the validation of a comprehensive ageism scale that aims to capture its three widely accepted core dimensions—stereotypes, prejudices, and discrimination—providing a more nuanced tool to assess ageism across diverse contexts and populations.

### What is ageism and why is it important?

Ageism is defined as prejudices, stereotypes, and discrimination towards people because of their chronological age [1]. It is manifested at the institutional level in rules, regulations, and policies that may prevent access to goods and services based on one’s chronological age [2]. Ageism also appears in interpersonal relations. It occurs when people judge others as too old or too young to do something simply because of their age, or when they behave according to age-related stereotypes—for example, by avoiding people of certain age groups or treating them in patronising ways [3]. At the intrapersonal level, ageism is directed towards oneself, with older persons internalising negative messages about age and ageing throughout their lives and self-directing these stereotypes and translating them into views of oneself as an older person when they become older [4–7].

Ageism is highly prevalent, with 1 in 3 people reporting the experience of ageism [8] and 1 in 2 people reporting being ageist [9]. It is manifested in a variety of settings and contexts including the health care system [10], the workplace [11, 12], digital technology [13], climate change discourse [14, 15], and the beauty industry [15]. Although ageism can also be directed towards younger individuals [16], most empirical research on its negative effects has focused on older persons [3]. Ageism directed towards oneself has shown to be related to reduced health and wellbeing, greater physical impairment, reduced ability to recover from surgery, a higher likelihood of falls, and reduced life expectancy [17–19]. Ageism directed towards older persons by others has also been shown to negatively impact health and wellbeing consequences, with older persons experiencing impaired cognitive functioning, physical health, and even depressive symptoms following exposure to perceived age-based discrimination [10]. The financial impact of ageism is notable as well. A recent study has estimated the impact of ageism in the U.S. healthcare system at $63 billion annually [20]. The financial impact of ageism in the U.S. workforce was estimated at $850 billion annually [21].

### The need for a new measure of ageism

Considering the negative effects of ageism on older persons and society at large, the World Health Organization (WHO) has embarked on a global campaign to combat ageism starting in 2016, with the expectation that it will take at least 15 years to create a societal change [22]. As part of the global campaign, the WHO launched the first ever global report on ageism. The report, which was based on several systematic reviews concerning the nature, determinants, impact, measurement, and consequences of ageism concluded that one of the tasks faced by the research community is the development and psychometric validation of a new measure to assess ageism.

The recommendation to develop a new measure to assess ageism was inspired by a systematic review which examined the psychometric properties of 11 existing ageism scales [23]. The review found that only one scale met minimum requirements for psychometric validation, consisting of content validity, structural validity, and internal consistency. However, the scale only captured the stereotype dimension of ageism. As the most contemporary definition of ageism includes three dimensions manifested as *prejudices* (e.g. feelings towards older persons), *stereotypes* (e.g. thoughts and beliefs about older persons), and *discrimination* (e.g. behaviours directed towards older persons) [1], it is important to develop a scale, which adequately captures and distinguishes between the three dimensions. Such a scale is needed to capture the scope and magnitude of ageism across different cultures and countries and to assess progress in the fight against ageism. The distinction along the three dimensions of ageism is important for conceptual reasons [1]. Moreover, such a distinction is also empirically relevant. For instance, current evidence in the fight against ageism suggests that interventions can improve attitudes towards older persons and increase comfort in interacting with them, but they have no significant effect on reducing anxiety or increasing willingness to work with older persons [24]. These findings highlight that interventions affect different dimensions of ageism in varying ways, which can only be effectively captured through an innovative measure that captures all three dimensions. Such a Standards for the selection of health Measurement INstruments guidelines [25].

### The present study

This study aimed to validate an innovative measure to assess ageism towards older persons from the perspective of the agent (the perpetrator) of ageism. The new measure should capture all three dimensions of ageism: prejudices, stereotypes, and discrimination. The target population of the new measure includes adults 20 years and older, encompassing the entire adult lifespan, as older persons can also engage in ageism directed towards other older persons [26]. To ensure cross-cultural validity, the sample originates from four countries, the Czech Republic [CZ], Germany [DE], Israel [IL], and the United Kingdom [UK].

The testing of the new measure involved developing a conceptualisation for the scale and creating an item-pool with input from academics, practitioners, policy experts, and older persons from diverse continents and countries, as well as evaluating the content validity of the items. These steps are reported elsewhere [27]. The present study reports on the structural validity, measurement invariance, internal consistency, and construct validity of the new WHO Ageism Towards Older Persons Scale to measure ageism directed towards older persons. This complements the previously validated WHO Ageism Experiences Scale, which draws on the same item pool but focuses on respondents’ experiences of ageism targeted towards them (including self-directed ageism) [28]. Given the conceptual definition of ageism as composed of three dimensions—prejudice, stereotypes, and discrimination [1]—we ensured that items tap into all three dimensions.

The four included countries represent diverse welfare regimes, varying proportions of older persons in their population, and different levels of ageism reported by both young and older individuals [29–31], in addition to using different languages. We started by establishing the structural validity of the new measure first within each country and then across countries, based on the expectation that a measure used in different cultures and settings should yield comparable results across diverse groups. Measurement invariance testing (e.g. the ability to interpret the measure in the same way) across countries was made possible because the four different countries that took part in the data collection employed comparable methods.

We also examined invariance across different age groups (20–45, 46–65, 66+). This approach is grounded in the lifespan perspective, which views age and ageing as qualitatively different at various stages of life [32]. Hence, to better understand ageism, it is important to assess how it manifests across the lifespan, rather than assume that only younger persons can be agents of ageism. In addition, we anticipated that the measure would correlate with previously validated scales measuring prejudice, stereotypes, and discrimination related to older age, as it aims to capture the multifaceted nature of ageism. Conversely, we hypothesised that it would be uncorrelated or negatively correlated with feelings towards the middle-aged (40s–50s) and younger age groups (20s–30s), since prejudice against older persons is not typically extended to these groups [16]. Likewise, we expected the new measure to be uncorrelated with the number of friends under 30, as ageist discrimination does not typically correlate with relationships with younger individuals. Finally, given the inconclusive results of prior research [33], we did not form specific hypotheses about age differences for the new measure, leaving this as an exploratory aspect for investigation. Likewise, no specific hypotheses were formed concerning the relationship between the experiences of ageism and the perpetration of ageism.

See Appendix 1 in the Supplementary Data section for the full details of the methods section, including participants, measures, and statistical procedure.

## Results

### Descriptive analyses

Descriptive statistics for all WHO-A-TOPS items are presented in Table 1. Responses generally indicated low levels of ageism, as reflected by mean scores below 2.5 on most items (response scale: 1–5). The sample consisted of participants from four countries: Czech Republic (*n* = 338), Germany (*n* = 391), Israel (*n* = 346), and the United Kingdom (*n* = 694).

**Table 1:** Descriptive statistics for the full set of WHO-A-TOPS items (retained items are indicated in bold)

Item	Item Dimension and Number	*N*	*M*	*SD*	min	max
**Older adults have a lot to contribute to society.**	Stereotype_1	1759	1.84	0.81	1	5
Older adults should stick to being around people their own age.^R^	Stereotype_2	1759	2.18	0.97	1	5
Older adults are too old for romance.^R^	Stereotype_3	1759	1.81	0.91	1	5
**Older adults are a burden.** ^**R**^	Stereotype_4	1758	1.82	0.91	1	5
It is worthwhile investing resources in older adults.	Stereotype_5	1758	1.99	0.87	1	5
Older adults are too old to change.^R^	Stereotype_6	1758	2.55	1.14	1	5
Older adults are capable of using technology.	Stereotype_7	1759	2.13	0.91	1	5
**I feel comfortable around older adults.**	Prejudice_1	1757	2.05	0.85	1	5
**I feel frustrated with older adults.** ^**R**^	Prejudice_2	1755	2.13	1.01	1	5
**I feel bored listening to older adults.** ^**R**^	Prejudice_3	1757	1.96	0.89	1	4
I feel pity for older adults.^R^	Prejudice_4	1757	2.77	1.19	1	5
**I enjoy being around older adults.**	Prejudice_5	1758	2.32	0.83	1	5
**I find older adults interesting.**	Prejudice_6	1758	2.07	0.80	2	5
I make jokes about older adults.^R^	Discrimination_1	1753	1.90	1.03	1	5
I talk to older adults in simplified language.^R^	Discrimination_2	1754	2.61	1.25	1	5
I exclude older adults from certain conversations.^R^	Discrimination_3	1748	2.09	1.07	1	5
**I avoid spending time with older adults.** ^**R**^	Discrimination_4	1753	1.93	0.92	1	4
**I listen to older adults.**	Discrimination_5	1756	1.95	0.71	1	5
**I ask older adults for their view.**	Discrimination_6	1754	2.10	0.82	1	5

*Note. M* and *SD* are used to represent mean and standard deviation, respectively. Items are scored from 1 = strongly agree to 5 = strongly disagree. R = reverse coded items. Higher scores reflect greater levels of reported ageism. Items were excluded iteratively based on criteria from Brown (2015): specifically, those with high residual variance (> 0.70) or low factor loadings (< 0.40), indicating poor model fit. This process ensured that only reliable and valid items were retained.

### Measurement invariance

The proposed three-factor model could not be successfully fitted across all countries. Model estimation revealed excessively high covariances between latent factors—exceeding one in some cases—indicating potential factor redundancy. These findings suggest that the proposed three-factor structure may not represent empirically distinct constructs within the sample. The substantial covariances between the latent factors indicated that a more parsimonious solution might better represent the data structure. We then proceeded with item selection through an iterative process. Specifically, items were excluded if they exhibited high residual variance (i.e. > 0.70) or low factor loadings (i.e. < 0.40), indicating poor model fit [34].

The resulting one-factor model, which included 10 items (see Table 1 for a detailed description of the items), achieved partial scalar measurement invariance across the four countries and demonstrated acceptable fit: χ^2^(158) = 473.129, CFI = 0.938, RMSEA = 0.076, SRMR = 0.067. Changes in the Comparative Fit Index (CFI), in the Root Mean Square Error of Approximation (RMSEA), and in the Standardised Root Mean Square Residual (SRMR) remained within the suggested limits [35]. For detailed results of the measurement invariance analyses across countries, see Table 2.

**Table 2:** WHO-A-TOPS: Multigroup CFA results. Global fit measures for the exact measurement equivalence of the 10-item model, countries: Czech Republic, Germany, Israel, and the United Kingdom

	Chi2(df)	RMSEA	CFI	SRMR
Configural	313.712 (116)^***^	0.071	0.961	0.044
Metric	399.871 (143)^***^	0.073	0.949	0.067
partial metric	373.748 (140)^***^	0.070	0.954	0.062
Scalar	713.864 (167)^***^	0.097	0.893	0.088
partial scalar	473.129 (158)^***^	0.076	0.938	0.067

*Note.* CFA = confirmatory factor analysis; RMSEA = root-mean-square error of approximation; CFI = comparative fit index; *SRMR =* standardised root-mean-square residual*.*

^***^ *P* < .001.

With respect to age groups, the resulting one-factor model achieved scalar measurement invariance and demonstrated acceptable fit: χ^2^(123) = 309.445, CFI = 0.963, RMSEA = 0.056, SRMR = 0.046. Changes in the CFI, in the RMSEA, and the SRMR remained within the suggested threshold limits [35]. For detailed results of the measurement invariance analyses across age groups, see Table 3. In summary, these results of measurement invariance analyses indicated the ability to capture a common construct across the four investigated countries and the three age groups.

**Table 3:** WHO-A-TOPS: Multigroup CFA results. Global fit measures for the exact measurement equivalence of the 10-item model, age groups: 20–45 years, 46–65 years, and 66+ years

	Chi2(df)	RMSEA	CFI	SRMR
Configural	236.739 (87)^***^	0.061	0.968	0.037
Metric	252.950 (105)^***^	0.055	0.969	0.042
Scalar	309.445 (123)^***^	0.056	0.963	0.046

*Note.* CFA = confirmatory factor analysis; RMSEA = root-mean-square error of approximation; CFI = comparative fit index; SRMR *=* standardised root-mean-square residual.

^^***^^ *P* < .001*.*

For further descriptive statistics, composite reliability, concurrent validity, cross-country, and age-related differences in the WHO-A-TOPS, see Appendix 2 in the Supplementary Data section. Appendix 3 features results for the two-factor solution.

## Discussion

The new tool represents an exceptional attempt to develop a measure of high psychometric properties following current state of the art guidelines [25], whilst taking into account the multidimensional nature of ageism [1]. The fact that four different countries, which represent different geographic and cultural regions participated in the study and that the measure was administered across the entire adult lifespan are additional advantages that should be stressed. The latter contribution is particularly notable in light of the fact that older persons are not only the subjects of ageism perpetrated by younger persons, but may also be the agents of ageism directed towards other older persons [32, 36]. Furthermore, our analysis ensures not only that the new measure is reliable and valid, but also that the same construct is being measured across different countries and cultures as well as across different age groups.

The new measure consists of 10 items designed to capture all three dimensions of ageism: stereotypes, prejudices, and discrimination. Contrary to expectations, the items do not cluster into three distinct dimensions. Instead, they are best described as reflecting a single underlying factor of ageism that incorporates all three dimensions without clearly distinguishing amongst them. However, a two-factor solution was empirically supported as well, though its psychometric properties were somewhat inferior. Researchers may thus choose to use the new scale either to capture stereotypes as distinct from prejudices and discrimination or as a wholistic measure of ageism. The single factor solution was supported not only within each country, but also by measurement invariance across countries and age groups. Although we established only partial scalar invariance for country comparisons, rather than full scalar invariance (which was established for age group comparisons), this still allows researchers to compare the overall mean of the new measure across countries, though the meaning of the different items might slightly vary across countries [37]. Moreover, the correlation patterns obtained supported the validity of the new measure, which showed correlations with all measures of ageism that were assessed in this study, and demonstrated meaningful relations to correlates of ageism (e.g. number of friends). Interestingly, the new measure showed positive correlations with age norms prescribing disengagement but showed negative correlations with expectations that older persons should be active and engaged.

In contrast to expectations, the new measure is unable to empirically distinguish between the three dimensions of ageism. There are two possible reasons for this: First, it could be argued that although stereotypes, prejudice, and discrimination can be conceptually distinguished, and capture different facets of ageism (e.g. [1]), these facets mutually influence each other, which leads to high correlations that prevent a clear factorial separation [38]. For instance, age stereotypes have been argued to influence and bias evaluative judgements of older people (i.e. prejudice), which in turn triggers negative behaviours like distancing from or excluding older people (i.e. age discrimination). Similarly, age discriminating behaviours might be justified by drawing on negative stereotypes or prejudice. A second argument for the difficulty to distinguish between stereotypes, prejudice and discrimination is that the conceptual borders between the three facets are somewhat blurred. Stereotypical beliefs involving attributes that have a clear valence (e.g. older people are rigid and conservative, and they stand in the way of necessary societal change) are hard to distinguish from evaluative prejudice against older people, and these negative stereotypes and evaluations in turn are expressed through behaviours that aim at reducing the influence of older people, or excluding them from public discourse and decision making [39]. The conceptual overlap between the three facets of ageism also becomes apparent when investigating the items that capture these facets, which use the term ‘prejudice’ interchangeably. For instance, to assess ageism as part of the European Social Survey, individuals are queried: ‘How often has anyone shown prejudice against you or treated you unfairly because of your age?’ [40]. Hence, although the term prejudice is not consistently or explicitly defined across the questions, its use suggests ambiguity in how the concept is understood. Hence, addressing ageism as a multidimensional construct, rather than attempting to differentiate each of its dimensions is a more viable option.

### Limitations

The present study has several limitations that should be noted. First, although we employed comparable strategies for data collection, they were not identical, with Israel and the UK relying on a somewhat different order of survey items. Moreover, none of the countries relied on representative, probability samples. In addition, although the original pool of items was developed by a highly diverse group of experts and lay people [27], it did not include representatives from all four countries that participated in this study. This may leave some room for cultural nuances, which were not captured in the development of the measure. We also could not distinguish any non-invariance due to language vs. culture because in each country only a single language of administration was employed. Hence, further research is needed to fully establish the scale’s factor-solution in addition to exploring other psychometric properties such as sensitivity to intervention effects and test–retest reliability. As younger persons also are the targets of ageism, it is important to validate a scale which measures perceived exposure to ageism in younger age groups. In addition, our sampling frame was largely Euro-centred (except for Israel). Future research will benefit from testing the psychometric properties of the new measure in the developing world, including Africa, Asia, and South America.

It also is worth noting that, despite our attempt to identify an equal number of items which represent each of the three domains of ageism, we ended up with a relatively large number of items that capture prejudice and a small number of items that capture stereotypes. This discrepancy further highlights the gap between theory and empirical findings. Moreover, had the study begun with a broader pool of potential items, it is possible that a different set of items, i.e. a different configuration, might have emerged. Yet, our decision to administer only 6–7 items per subscale and reduce the number of items based on empirical evidence was derived by a need for balance between our wish for comprehensiveness and our attempt not to burden respondents by asking them to respond to too many items. Nonetheless, it is important to emphasise that all three dimensions of ageism are captured by the new measure. Since it is recommended for use as a single factor, the exact number of items representing each domain becomes less crucial.

## Conclusion

This study is a major milestone in ageism research as it provides, for the first time, a psychometrically valid and reliable measure which can be used across different countries and age groups. Moreover, the present study also can serve as a gold standard for future development of new measures, given its robust methodology. To capture ageism directed towards older persons across the lifespan, we recommend that researchers use the new measure either as a single-factor scale or as a two-factor scale. We recommend usage of the two-factor solution if researchers want to investigate specific hypotheses relating to either stereotypes or prejudice/discrimination, or to differences between the two components, but we recommend the single-factor scale as a default solution due to its superior psychometric properties, keeping in mind the fact that all three dimensions of ageism are captured by the 10 items.

## Supplementary Material

aa_25_2278_file002_afaf384_LA
