# Correction to: The World Health Organization ageism towards older persons scale: preliminary validation of a novel measure of ageist stereotypes, prejudices, and discrimination in four different countries

**DOI:** 10.1093/ageing/afag067

**Published:** 2026-03-16

This is a correction to: Liat Ayalon, M Clara Maria P de Paula Couto, Klaus Rothermund, Jana Nikitin, Xuefei Li, Zhuoni Xiao, Aja Louise Murray, The World Health Organization ageism towards older persons scale: preliminary validation of a novel measure of ageist stereotypes, prejudices, and discrimination in four different countries, *Age and Ageing*, Volume 55, Issue 1, January 2026, https://doi.org/10.1093/ageing/afaf384

In the originally published version of the paper there were errors within the numbering of references with the supplement data file. These have been corrected.

