# Supplementary material for: The World Health Organization ageism towards older persons scale: preliminary validation of a novel measure of ageist stereotypes, prejudices, and discrimination in four different countries
Source: Age Ageing. 2026 Jan 24;55(1):afaf384. doi: 10.1093/ageing/afaf384 (PMC12831184; doi:10.1093/ageing/afaf384)
Supplement: aa_25_2278_file002_afaf384_LA [file aa_25_2278_file002_afaf384_la.docx]

**The WHO Ageism Towards Older Persons Scale (WHO-A-TOPS): Preliminary Validation of a Novel Measure of Ageist Stereotypes, Prejudices, and Discrimination in Four Different Countries**

Appendix 1

**Methods**

**Participants**

Participants were recruited in four different countries: the Czech Republic [CZ], Germany [DE], Israel [IL], and the United Kingdom [UK] using similar procedures and comparable inclusion and exclusion criteria. Each of the four samples was recruited online relying on local market research institutes to administer the survey. Participants were native speakers of the main language in each country and at least 20 years old. They completed the study in exchange for money, at a price estimated based on expected average completion time. The sample was stratified by age (20-45, 46-65, 66+ years) and gender (50% female). Data were collected between September 2024 and March 2025. All respondents provided informed consent prior to their participation in the study. The study was approved by a University Ethics Committee in each of the four countries (for CZ: University of Vienna, Institutional Review Board of the Department of for Developmental and Educational Psychology, Austria: #08_25, Friedrich Schiller University, Germany: FSV 18/36; Bar Ilan University, Israel: #032506; and University of Edinburg, UK: #388-2324/9)

Table S1 provides a detailed description of the four samples. In total, 1,778 participants (*M*age = 51.9 years, *SD* = 17.0, age range 20 to 90 years, 50.5% female) completed the survey. For the main analysis of this study, we excluded 23 participants based on data quality concerns. Nine participants failed both attention check items embedded in the scale, and another nine showed no response variability across the newly developed ageism scale (i.e., provided the same answer to every item), suggesting a lack of thoughtful engagement. As such patterns may compromise data validity and skew results (e.g., [1]), these cases were removed. Additionally, five participants failed all attention checks and also exhibited no response variability and were hence excluded as well. The final sample was therefore *N* = 1,759.

**Measures**

All measures were originally developed in English and translated into Czech, German, and Hebrew in accordance with the WHO translation guidance [2]. This process, known as TRAPD (Translation, Review, Adjudication, Pretest, and Documentation), is considered the gold standard for survey translation. It involves independent translations by at least two native speakers of each target language, followed by a review and reconciliation of discrepancies through discussion.

***The WHO Ageism Towards Older Persons Scale (WHO-A-TOPS)***

Items for the WHO Ageism Towards Older Persons Scale (WHO-A-TOPS) were selected from the WHO ageism item pool [3]. Three experts in the field of gerontology and one expert in psychometrics— each representing one of the four participating countries — independently reviewed the items. They then discussed their selections collectively to reach a consensus.

To ensure adequate coverage of key content areas while maintaining a sufficiently brief scale that would be feasible to administer without placing undue burden on participants, the scale was intended to include 9 to 15 items in total, with 3 to 5 items dedicated to each construct—age-based stereotypes, prejudice, and discrimination. However, a larger pool of 19 items was selected for an initial stage of data collection (see Table 1 in the main text for a detailed description of the items). Items were rated on a 5-point Likert scale ranging from 1 (*strongly agree*) to 5 (*strongly disagree*). Participants were also given a ‘don’t know or not applicable’ option, which was treated as missing data for the present analyses. To compute composite scores, negatively keyed items (e.g., *“Older adults are a burden”*; *“I feel frustrated with older adults”*; *“I avoid spending time with older adults”*) were reverse-coded. Item responses were then averaged so that higher scores reflected greater ageism towards older persons.

To validate the WHO-A-TOPS scale, we assessed convergent and discriminant validity using several measures, as detailed below:

***Descriptive Age Stereotypes***

We used the Aging Semantic Differential [4] to assess descriptive age stereotypes. In the ASD, participants use a 7-point scale to rate 32 pairs of bipolar adjectives (e.g., *“in my personal opinion older adults are...”*, 1 = *“healthy”/ “optimistic”*, 7 = *“unhealthy”/ “pessimistic”*). Lower scores represent a more positive attitude toward older persons on the respective adjective pair (Cronbach’s α = .98).

***Prescriptive Age Stereotypes***

Endorsement of prescriptive age stereotypes was assessed with a 7-item scale [5] that includes three items capturing altruistic disengagement (e.g., *“In my personal opinion, older adults should make way for the younger generation by giving up important roles (e.g., at work, in politics).”*) and four items capturing active aging (e.g., *“In my personal opinion, older adults should contribute to society as long as possible.”*). Respondents were asked to rate the items on a 5-point Likert scale ranging from 1 (*do not agree*) to 5 (*strongly agree*). Higher values in the scales reflect greater endorsement of altruistic disengagement and active aging. Cronbach’s α for altruistic disengagement was .51 and for active aging .78.

***Age-Based Prejudice***

Respondents were asked to rate their feelings toward different age groups, ranging from individuals in their 20s to those in their 90s. They answered the question, *“Please share how you feel toward different age groups,”* using a scale from 0 (*extremely negative feelings*) to 10 (*extremely positive feelings*). A similar measure has previously been used in the European Social Survey [6]. To compute a score reflecting prejudice toward older persons, we first averaged participants’ responses for the younger age groups (20 to 50 years) and for the older age groups (60 to 90 years). We then subtracted the average for the younger age groups from the average for the older age groups. Negative scores indicate relatively more positive feelings toward younger persons compared to older persons.

***Age-Based Stereotype Threat***

**Age-Based Stereotype Threat (ABST)**was assessed using a domain-specific scale adapted from [7]. The adapted scale captures ABST across seven domains: work, physical activity, driving or using public transportation, learning or using new technology, leisure activities, engaging with the healthcare system or healthcare professionals, and appearance. For each domain, participants responded to four items assessing: (1) awareness of their age, (2) concern that others might judge them because of their age, (3) fear of confirming a negative age stereotype, and (4) avoidance of situations where they might be judged based on age. Items were rated on a 5-point scale ranging from 1 (**never**) to 5 (**very often**). Cronbach’s α for the scale was .96.

***Age Discrimination***

We used the Relating to Older People Evaluation (ROPE) scale [8] to assess age-based discrimination toward older persons. The ROPE is a 20-item scale, with responses given on a 3-point scale ranging from 1 (*never*) to 3 (*often*). The measure includes six positive items (e.g., *“Hold doors open for old people because of their age.”*) and 14 items that reflect negative aspects of ageism (e.g. *“Ignore old people because of their age.”*). Reliability for the positive and negative items was acceptable across the four examined countries (Cronbach’s α for positive ageism = .65, and for negative ageism = .73).

***Intergenerational Relations***

Intergenerational relations were examined by the following two items [6]: *“About how many friends, other than members of your family, do you have who are younger than 30/older than 70?”.*

***WHO Ageism Experiences Scale***

This scale is a 15-item measure of perceived ageism experiences from the target’s perspective [9]. The measure addresses three facets of ageism experiences: (1) self-directed ageism (e.g., *“I am a burden because of my age.”*), (2) other-directed ageism (e.g., *“Others make decisions for me because of my age.”*), and (3) institutionalized ageism *(“I have been turned down for an opportunity e.g., a job or volunteering opportunity that I was qualified for because of my age.”*). Responses were given on a 5-point Likert scale ranging from 1 (*strongly agree*) to 5 (*strongly disagree*), with an extra option *‘do not know or not applicable’*, which was coded as missing. Negatively keyed items were reverse coded such that a higher score in this measure indicates higher levels of perceived ageism experiences. Cronbach’s α for the scale was .84.

***Sociodemographic Information***

We asked participants about their age (in years), gender (1 = *female*, 2 = *male*, 3 = *prefer not to say*), and their education level (in years).

**Statistical Procedure**

***Measurement Invariance***

To address potential concerns regarding contextual and age-group specificity in the construct of ageism toward older persons as measured by the WHO-A-TOPS, we conducted multiple-group confirmatory factor analyses (MG-CFA) and tested for measurement invariance. A three-factor model of the WHO-A-TOPS, using all 19 items, was first evaluated across countries and then across age groups. All models were estimated using robust maximum likelihood (MLR), and missing data were handled using Full Information Maximum Likelihood (FIML) as implemented in the *lavaan* package [10].

Measurement invariance was examined in a stepwise fashion—configural, metric, and scalar. When full invariance could not be established, we closely examined the modification indices (MI > 10) and expected parameter changes (EPC > .20) to identify the most non-invariant parameters at each step. These parameters were then progressively freed to assess partial invariance. Partial invariance was considered **acceptable if at least two loadings or intercepts per factor remained invariant and the number of freely estimated parameters did not exceed 25-30% [11]** . Model comparisons were evaluated using recommended criteria for changes in the Comparative Fit Index (CFI), Root Mean Square Error of Approximation (RMSEA), and Standardized Root Mean Square Residual (SRMR), as proposed by [12].

Moreover, items were excluded through an iterative process if they exhibited high residual variance (i.e., > .70) or low factor loadings (i.e., < .40), indicating poor model fit [13]. We first examined measurement invariance across the four countries included in this study (CZ, DE, IL, and UK). We then fitted the same model to test for measurement invariance across age groups (20-45 years, 46-65 years, 66+ years).

***Correlations and Analyses of Variance***

We assessed the construct validity of the new WHO-A-TOPS measure by examining its correlations with the various constructs included in this study. Given that the assumptions of normality and homogeneity of variance were not fully met for the WHO-A-TOPS scale, a nonparametric Kruskal–Wallis H test was conducted to compare expressions of ageism towards older persons across four countries and three age groups.

**References**

1. Osborne JW, Blanchard MR. Random responding from participants is a threat to the validity of social science research results. Front Psychol 2011;1:220. doi:10.3389/fpsyg.2010.002202
2. World Health Organization. Process of translation and adaptation of instruments. Geneva: World Health Organization; 2016. Available from: <https://www.who.int/publications/i/item/9789241512747> (accessed 02/14/2026)
3. Murray AJ, de la Fuente-Núñez V. Development of the item pool for the ‘WHO-ageism scale’: conceptualisation, item generation and content validity assessment. Age Ageing 2023;52(Suppl_4):iv149–iv157. doi:10.1093/ageing/afad1054
4. Rosencranz HA, McNevin TE. A factor analysis of attitudes toward the aged. Gerontologist 1969;9(1):55–59
5. de Paula Couto MC, Fung HH, Graf S, Hess TM, Liou S, Nikitin J, et al. Antecedents and consequences of endorsing prescriptive views of active aging and altruistic disengagement. Front Psychol 2022;13:807726. doi:10.3389/fpsyg.2022.807726
6. European Social Survey. Experiences and expressions of ageism: Module template with background information, survey questions. Available from: <https://www.europeansocialsurvey.org/sites/default/files/2023-06/ESS4_final_ageism_module_template.pdf> (accessed 08/03/2025)
7. Lamont RA, Swift HJ, Drury L. Understanding perceived age-based judgement as a precursor to age-based stereotype threat in everyday settings. Front Psychol 2021;12:640567. doi:10.3389/fpsyg.2021.640567
8. Cherry KE, Palmore E. Relating to older people evaluation (ROPE): a measure of self-reported ageism. Educ Gerontol 2008;34(10):849–61
9. Murray AL, Li X, Booth T. Preliminary validation of the 15-item WHO experiences of ageism scales in a mixed-age UK sample. 2025. OSF Preprint. <https://doi.org/10.31234/osf.io/7jcqk_v2> (accessed 11/01/2025)
10. Rosseel Y. lavaan: An R package for structural equation modeling. J Stat Softw 2012;48(2):1–36
11. Byrne BM, Shavelson RJ, Muthén B. Testing for the equivalence of factor covariance and mean structures: the issue of partial measurement invariance. Psychol Bull 1989;105(3):456–66
12. Chen FF. Sensitivity of goodness-of-fit indexes to lack of measurement invariance. Struct Equ Modeling 2007;14(3):464–504
13. Brown TA. Confirmatory factor analysis for applied research. 2nd ed. New York: Guilford Press; 2015

Appendix 2

**Results**

Composite reliability (CR) was computed using Raykov’s method [1] to assess the internal consistency of the latent construct (i.e., expressions of ageism towards older persons) across countries. The CR values indicated acceptable to good reliability: Germany (CR = 0.80), Czech Republic (CR = 0.73), Israel (CR = 0.82), and the United Kingdom (CR = 0.77). All values met or exceeded the commonly recommended threshold of 0.70 [2], indicating satisfactory construct reliability across all countries.

Regarding age groups, CR values were similarly acceptable and consistent: 66+ years (CR = 0.78), 46–65 years (CR = 0.79), and 20–45 years (CR = 0.79). As with the country comparisons, all values exceeded the 0.70 threshold, supporting satisfactory reliability across age groups.^[[1]](#footnote-1)^

**Concurrent Validity Correlations**

See Table S2 for sample size, means, standard deviations, and correlations between study variables with respect to the one-factor solution. WHO-A-TOPS (Expressions of Ageism Towards Older Persons) was significantly and positively correlated with measures of negative age-related attitudes, including the Aging Semantic Differential (ASD; *r* = .32, *p* < .01), Age-Based Stereotype Threat (ABST; *r* = .18, *p* < .01), and prescriptive age stereotypes related to Altruistic Disengagement (DIS; *r* = .23, *p* < .01). It was also positively associated with Negative Ageism (ROPE_N; *r* = .15, *p* < .01) and experiences of ageism (WHO; *r* = .29, *p* < .01). Conversely, WHO-A-TOPS scores were negatively correlated with Positive Ageism (ROPE_P; *r* = –.40, *p* < .01) and prescriptive age stereotypes related to Activation (ACT; *r* = –.17, *p* < .01), indicating that higher ageism was linked to fewer positive ageist behaviors and lower endorsement of active aging prescriptive beliefs. A positive correlation was observed with relative evaluations favoring younger adults (EVAL; *r* = .33, *p* < .01), while correlations with intergenerational contact were nonsignificant or small (IC_30: *r* = .03; IC_70: *r* = –.14, *p* < .01). These findings support the concurrent validity of the WHO-A-TOPS measure.

**Cross-Country Differences in Expressions of Ageism Towards Older Persons**

A Kruskal–Wallis H test indicated a statistically significant difference in expressions of ageism towards older persons (WHO-A-TOPS) across countries, χ²(3) = 53.14, *p* < .001. Follow-up pairwise Wilcoxon rank sum tests with Holm-adjusted *p*-values showed that participants from the Czech Republic reported significantly higher ageism scores than those from Germany (*p* < .001), Israel (*p* < .001), and the United Kingdom (*p* < .001). No significant differences were observed among Germany, Israel, and the United Kingdom (all *p*s > .80). Descriptive statistics revealed that the Czech Republic had the highest mean ageism score (*M* = 2.21, *SD* = 0.53), followed by Germany (*M* = 2.00, *SD* = 0.60), Israel (*M* = 1.99, *SD* = 0.55), and the United Kingdom (*M* = 1.95, *SD* = 0.56). Figure S1 depicts the adjusted means of WHO-A-TOPS across the four examined countries and their pairwise comparisons.

**Age-Related Differences in Expressions of Ageism Towards Older Persons**

A Kruskal–Wallis H test revealed a statistically significant difference in expressions of ageism towards older persons (WHO-A-TOPS) across age groups, χ²(2) = 40.22, *p* < .001. Follow-up pairwise Wilcoxon rank sum tests with Holm-adjusted *p*-values indicated that participants aged 20–45 years reported significantly higher ageism scores than those aged 46–65 years (*p* < .001) and those aged 66 years and older (*p* < .001). No significant difference was found between the 46–65 and 66+ age groups (*p* = .40). Descriptive statistics showed that the 20–45 years group had the highest mean ageism score (*M* = 2.14, *SD* = 0.59), followed by the 66+ years group (*M* = 1.96, *SD* = 0.52) and the 46–65 years group (*M* = 1.94, *SD* = 0.57). Figure S2 depicts the adjusted means of WHO-A-TOPS across the four-examined countries and their pairwise comparisons.

| **Tables**  **Table S1**  *Sample Description by Country* | | | | | |
| --- | --- | --- | --- | --- | --- |
| **Characteristic** | **CZ** *N* = 338*^1^* | **DE** *N* = 391*^1^* | **IL** *N* = 346*^1^* | **UK** *N* = 694*^1^* | ***p*-value***^2^* |
| Sex |  |  |  |  | 0.953 |
| Female | 173 ^a^ (51.18%) | 194 ^a^ (49.62%) | 174 ^a^ (50.29%) | 356 ^a^ (51.30%) |  |
| Age (years) | 52.41 ^a^ (19.31) | 54.99 ^ab^ (16.02) | 50.34 ^ac^ (17.10) | 50.72 ^ac^ (16.01) | <0.001 |
| Education (years) | 14.17 ^a^ (3.49) | 13.69 ^a^ (4.51) | 14.30 ^a^ (2.58) | 15.73 ^b^ (3.74) | <0.001 |
| Age Group |  |  |  |  | 0.040 |
| 20-45 years | 116 ^a^ (34.32%) | 129 ^a^ (32.99%) | 132 ^a^ (38.15%) | 265 ^a^ (38.29%) |  |
|  | Age: 29.60 (7.75) | Age: 35.70 (6.50) | Age: 32.10 (7.40) | Age: 33.50 (7.04) |  |
| 46-65 years | 108 ^a^ (31.95%) | 135 ^a^ (34.53%) | 109 ^a^ (31.50%) | 254 ^a^ (36.71%) |  |
|  | Age: 54.40 (5.88) | Age: 57.10 (5.79) | Age: 52.70 (5.37) | Age: 55.30 (6.05) |  |
| 66+ years | 114 ^a^ (33.73%) | 127 ^ab^ (32.48%) | 105 ^ab^ (30.35%) | 173 ^b^ (25.00%) |  |
|  | Age: 73.70 (4.60) | Age: 72.40 (5.09) | Age: 70.90 (3.97) | Age: 70.50 (4.23) |  |
|  | Age range: 66-86 | Age range: 66-85 | Age range: 66-82 | Age range: 66-90 |  |
| *Note. ^1^*n (%); Mean (SD), *^2^*Pearson's Chi-squared test; Kruskal-Wallis rank sum test, CZ = Czech Republic, DE = Germany, IL = Israel, UK = United Kingdom | | | | | |

**Table S2**

*Sample Size, Means, Standard Deviations, and Correlations Between Study Variables (One-Factor Solution)*

| Variable | *N* | *M* | *SD* | 1 | 2 | 3 | 4 | 5 | 6 | 7 | 8 | 9 | 10 |
| --- | --- | --- | --- | --- | --- | --- | --- | --- | --- | --- | --- | --- | --- |
|  |  |  |  |  |  |  |  |  |  |  |  |  |  |
| 1. WHO-A-TOPS | 1,759 | 2.02 | 0.57 |  |  |  |  |  |  |  |  |  |  |
|  |  |  |  |  |  |  |  |  |  |  |  |  |  |
| 2. ASD | 1,026 | 2.98 | 1.21 | .32** |  |  |  |  |  |  |  |  |  |
|  |  |  |  |  |  |  |  |  |  |  |  |  |  |
| 3. ABST | 1,026 | 1.98 | 0.74 | .18** | .03 |  |  |  |  |  |  |  |  |
|  |  |  |  |  |  |  |  |  |  |  |  |  |  |
| 4. ACT | 1,727 | 3.64 | 0.83 | -.17** | -.00 | .06 |  |  |  |  |  |  |  |
|  |  |  |  |  |  |  |  |  |  |  |  |  |  |
| 5. DIS | 1,727 | 2.37 | 0.87 | .23** | .06* | .32** | .21** |  |  |  |  |  |  |
|  |  |  |  |  |  |  |  |  |  |  |  |  |  |
| 6. ROPE_P | 1,727 | 1.98 | 0.36 | -.40** | -.22** | .20** | .13** | .11** |  |  |  |  |  |
|  |  |  |  |  |  |  |  |  |  |  |  |  |  |
| 7. ROPE_N | 1,727 | 1.44 | 0.27 | .15** | .00 | .43** | .04 | .32** | .40** |  |  |  |  |
|  |  |  |  |  |  |  |  |  |  |  |  |  |  |
| 8. WHO | 1,420 | 2.06 | 0.58 | .29** | .15** | .61** | -.10** | .30** | .04 | .32** |  |  |  |
|  |  |  |  |  |  |  |  |  |  |  |  |  |  |
| 9. EVAL | 1,727 | -0.12 | 1.69 | .33** | .02 | .02 | -.01 | .12** | -.20** | .02 | -.01 |  |  |
|  |  |  |  |  |  |  |  |  |  |  |  |  |  |
| 10. IC_30 | 1,752 | 4.59 | 9.40 | .03 | -.03 | -.03 | -.12** | -.00 | .03 | .06* | .03 | .11** |  |
|  |  |  |  |  |  |  |  |  |  |  |  |  |  |
| 11. IC_70 | 1,750 | 3.68 | 6.91 | -.14** | -.09** | .05 | .15** | .03 | .17** | .08** | -.02 | -.07** | .12** |
|  |  |  |  |  |  |  |  |  |  |  |  |  |  |

*Note.* *M* and *SD* are used to represent mean and standard deviation, respectively. WHO-A-TOPS: Ageism towards older persons; ASD = Aging Semantic Differential; ABST = Age-based Stereotype Threat; ACT = Prescriptive age stereotypes – Activation; DIS = Prescriptive age stereotypes – Altruistic Disengagement; ROPE_P = Positive ageism; ROPE_N = Negative ageism; WHO =Ageism Experiences; EVAL = evaluations of younger relative to older adults. Positive values indicate more favourable evaluations of younger adults compared to older adults. IC_30 = number of friends 30 or younger; IC_70 = number of friends 70 or older. For all other variables, higher values indicate greater negativity: more ageism perpetrator, greater age-based stereotype threat, stronger endorsement of activation and altruistic disengagement, higher levels of ageism (as assessed by the ROPE and WHO scales), and more frequent contact with individuals aged 30 or younger / 70 or older. * *p* < .05. ** *p* < .01.

**Figures**

**Figure S1**

*Mean Adjusted WHO-A-TOPS Across Countries*

**
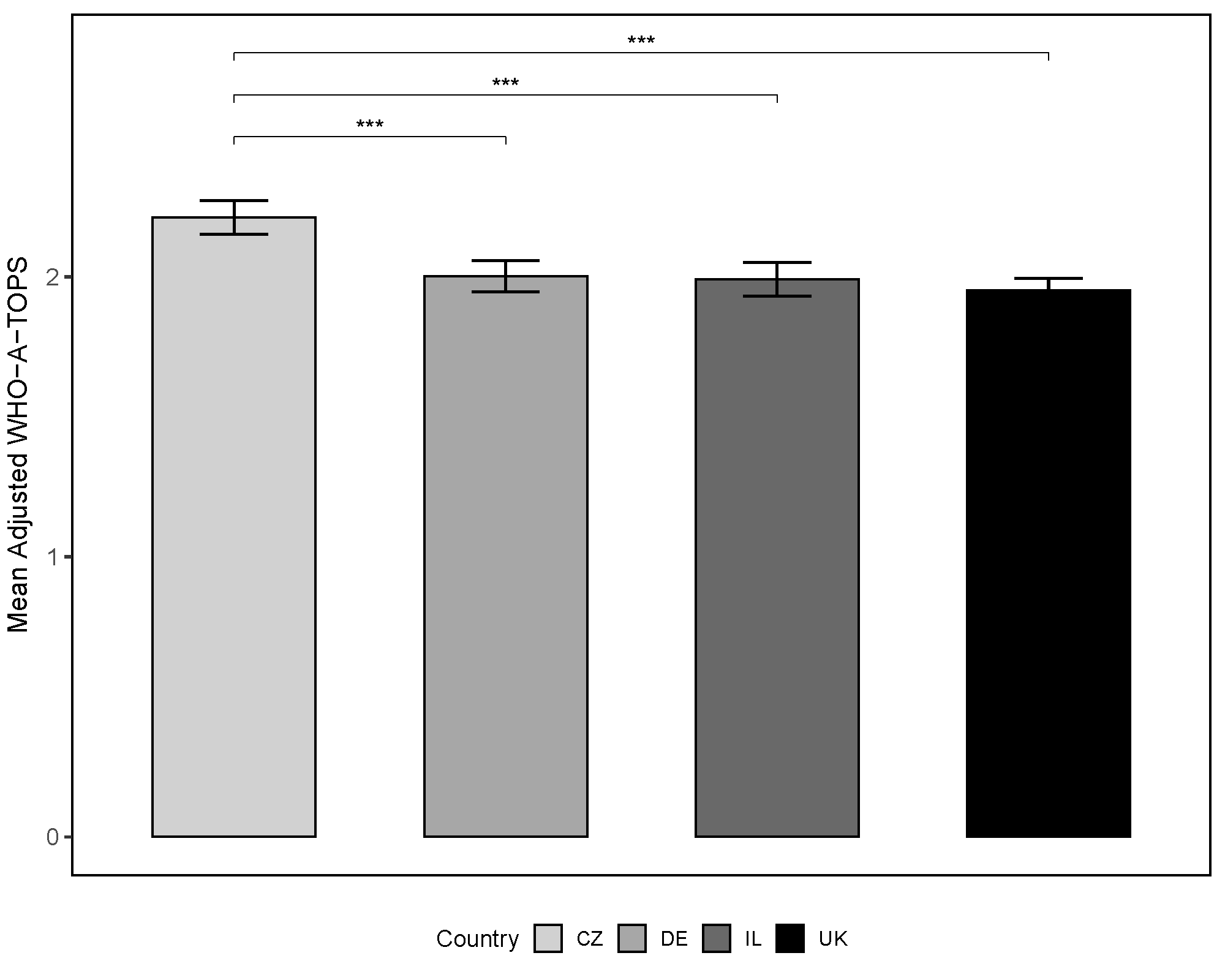
**

*Note.* Czech Republic (CZ), Germany (DE), Israel (IL), and the United Kingdom (UK).

*** indicates *p* < .001

**Figure S2**

*Mean Adjusted WHO-A-TOPS Across Age Groups*

**
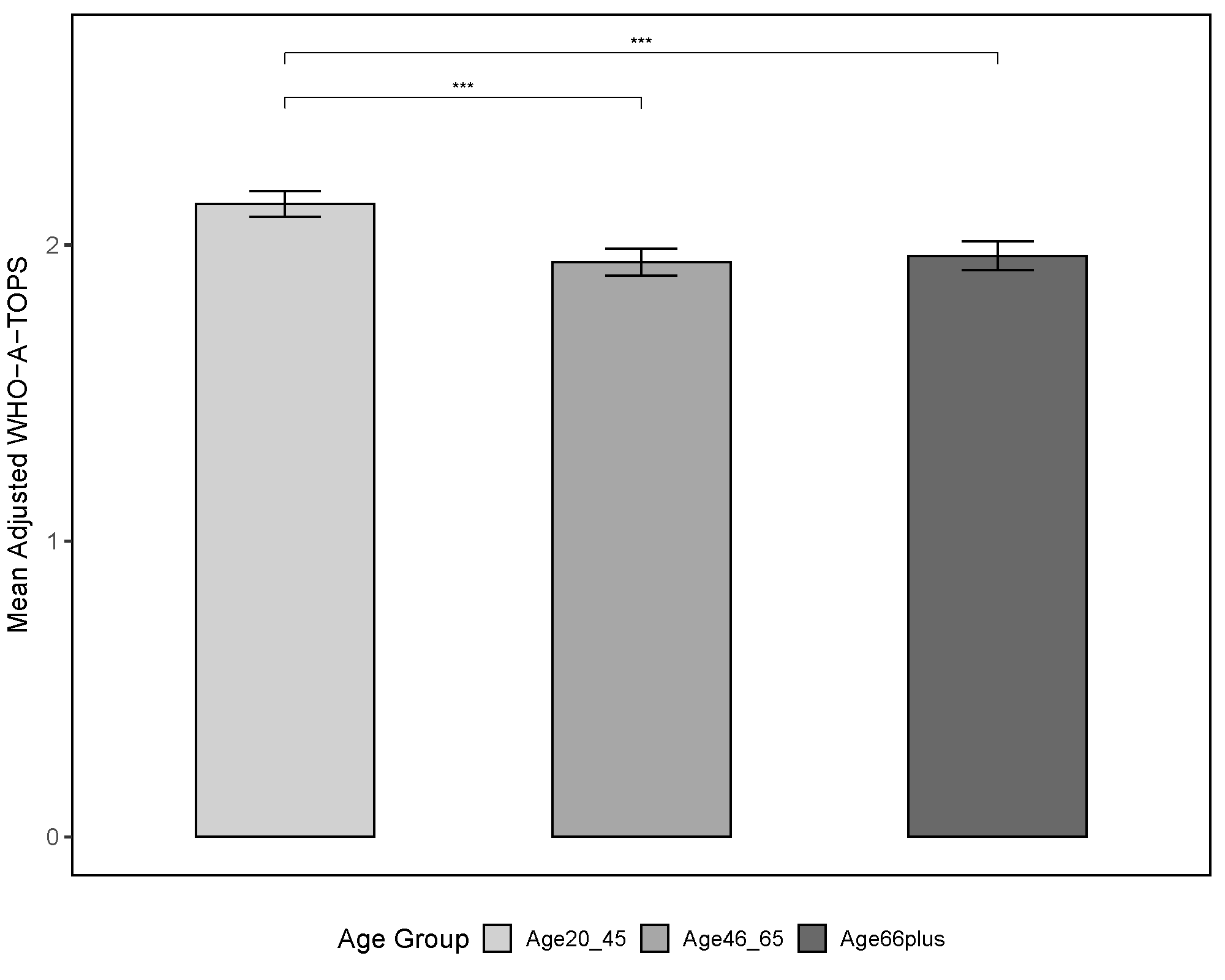
**

*Note.* *** indicates *p* < .001

**References**

1. Raykov T. Estimation of composite reliability for congeneric measures. Appl Psychol Meas 1997;21(2):173–184
2. Hair JF, Hult GTM, Ringle CM, Sarstedt M, Danks NP, Ray S. Evaluation of reflective measurement models. In: Partial Least Squares Structural Equation Modeling (PLS-SEM) using R: A workbook. Thousand Oaks, CA: Springer International Publishing; 2021. p.75–90

.

**Appendix 3**

**WHO-A-TOPS Two-Factor Solution of the WHO-A-TOPS Scale**

Table S3 presents descriptive statistics for the full set of WHO-A-TOPS items. Table S4 presents sample size, means, standard deviations, and correlations between study variables and the WHO-A-TOPS (Two-Factor Solution). Table S5 and S6 present the WHO-A-TOPS multigroup confirmatory factor analysis (CFA) across the four countries and across the three age groups: 20-45 years, 46-65 years, and 66+ years. Figures S3 and S4 present the mean adjusted WHO-A-TOPS for the two-tactor solution across countries and across age groups.

Composite reliability (CR) was computed using Raykov’s method [1] to assess the internal consistency of the latent constructs—expressions of ageism toward older persons, divided into prejudice/discrimination and stereotypes, respectively—across countries and age groups.

The CR values by country were as follows: Germany (CR = 0.89 for prejudice/discrimination and 0.46 for stereotypes), Czech Republic (CR = 0.84 and 0.48), Israel (CR = 0.80 and 0.39), and the United Kingdom (CR = 0.78 and 0.56).

For age groups, CR values were: 66+ years (CR = 0.82 and 0.52), 46–65 years (CR = 0.83 and 0.45), and 20–45 years (CR = 0.83 and 0.45).

**Tables**

**Table S3**

*Descriptive Statistics for the Full Set of WHO-A-TOPS Items (Retained Items for the Two-factor Solution are Indicated in Bold; Light Grey Represents the Stereotype Factor; Dark Grey Represents the Prejudice/Discrimination Factor)*

| **Item** | **Item Dimension and Number** | ***N*** | ***M*** | ***SD*** | **min** | **max** |
| --- | --- | --- | --- | --- | --- | --- |
| **Older adults have a lot to contribute to society.** | **Stereotype_1** | 1,759 | 1.84 | 0.81 | 1 | 5 |
| **Older adults should stick to being around people their own age. ^R^** | **Stereotype_2** | 1,759 | 2.18 | 0.97 | 1 | 5 |
| **Older adults are too old for romance. ^R^** | **Stereotype_3** | 1,759 | 1.81 | 0.91 | 1 | 5 |
| **Older adults are a burden. ^R^** | **Stereotype_4** | 1,758 | 1.82 | 0.91 | 1 | 5 |
| It is worthwhile investing resources in older adults. | Stereotype_5 | 1,758 | 1.99 | 0.87 | 1 | 5 |
| Older adults are too old to change**.** ^R^ | Stereotype_6 | 1,758 | 2.55 | 1.14 | 1 | 5 |
| Older adults are capable of using technology. | Stereotype_7 | 1,759 | 2.13 | 0.91 | 1 | 5 |
| **I feel comfortable around older adults.** | **Prejudice_1** | 1,757 | 2.05 | 0.85 | 1 | 5 |
| I feel frustrated with older adults. ^R^ | Prejudice_2 | 1,755 | 2.13 | 1.01 | 1 | 5 |
| **I feel bored listening to older adults. ^R^** | **Prejudice_3** | 1,757 | 1.96 | 0.89 | 1 | 4 |
| I feel pity for older adults. ^R^ | **Prejudice_4** | 1,757 | 2.77 | 1.19 | 1 | 5 |
| **I enjoy being around older adults.** | **Prejudice_5** | 1,758 | 2.32 | 0.83 | 1 | 5 |
| **I find older adults interesting.** | **Prejudice_6** | 1,758 | 2.07 | 0.80 | 2 | 5 |
| I make jokes about older adults. ^R^ | Discrimination_1 | 1,753 | 1.90 | 1.03 | 1 | 5 |
| I talk to older adults in simplified language. ^R^ | Discrimination_2 | 1,754 | 2.61 | 1.25 | 1 | 5 |
| I exclude older adults from certain conversations. ^R^ | Discrimination_3 | 1,748 | 2.09 | 1.07 | 1 | 5 |
| I avoid spending time with older adults. ^R^ | Discrimination_4 | 1,753 | 1.93 | 0.92 | 1 | 4 |
| **I listen to older adults.** | **Discrimination_5** | 1,756 | 1.95 | 0.71 | 1 | 5 |
| **I ask older adults for their view.** | **Discrimination_6** | 1,754 | 2.10 | 0.82 | 1 | 5 |

*Note. M* and *SD* are used to represent mean and standard deviation, respectively. Items are scored from 1=strongly agree to 5 = strongly disagree. R = reverse coded items. Higher scores reflect greater levels of reported ageism.

**Table S4**

*Sample Size, Means, Standard Deviations, and Correlations Between Study Variables (Two-Factor Solution)*

| Variable | *N* | *M* | *SD* | 1 | 2 | 3 | 4 | 5 | 6 | 7 | 8 | 9 | 10 | 11 |
| --- | --- | --- | --- | --- | --- | --- | --- | --- | --- | --- | --- | --- | --- | --- |
|  |  |  |  |  |  |  |  |  |  |  |  |  |  |  |
| 1. STER | 1,759 | 1.91 | 0.62 |  |  |  |  |  |  |  |  |  |  |  |
|  |  |  |  |  |  |  |  |  |  |  |  |  |  |  |
| 2. PR-DIS | 1,759 | 2.08 | 0.60 | **.54**** |  |  |  |  |  |  |  |  |  |  |
|  |  |  |  |  |  |  |  |  |  |  |  |  |  |  |
| 3. ASD | 1,026 | 2.98 | 1.21 | .20** | .34** |  |  |  |  |  |  |  |  |  |
|  |  |  |  |  |  |  |  |  |  |  |  |  |  |  |
| 4. ABST | 1,026 | 1.98 | 0.74 | .32** | .10** | .03 |  |  |  |  |  |  |  |  |
|  |  |  |  |  |  |  |  |  |  |  |  |  |  |  |
| 5. ACT | 1,727 | 3.64 | 0.83 | -.12** | -.17** | -.00 | .06 |  |  |  |  |  |  |  |
|  |  |  |  |  |  |  |  |  |  |  |  |  |  |  |
| 6. DIS | 1,727 | 2.37 | 0.87 | .36** | .18** | .06* | .32** | .21** |  |  |  |  |  |  |
|  |  |  |  |  |  |  |  |  |  |  |  |  |  |  |
| 7. ROPE_P | 1,727 | 1.98 | 0.36 | -.12** | -.45** | -.22** | .20** | .13** | .11** |  |  |  |  |  |
|  |  |  |  |  |  |  |  |  |  |  |  |  |  |  |
| 8. ROPE_N | 1,727 | 1.44 | 0.27 | .31** | .08** | .00 | .43** | .04 | .32** | .40** |  |  |  |  |
|  |  |  |  |  |  |  |  |  |  |  |  |  |  |  |
| 9. WHO | 1,420 | 2.06 | 0.58 | .36** | .22** | .15** | .61** | -.10** | .30** | .04 | .32** |  |  |  |
|  |  |  |  |  |  |  |  |  |  |  |  |  |  |  |
| 10. EVAL | 1,727 | -0.12 | 1.69 | .22** | .31** | .02 | .02 | -.01 | .12** | -.20** | .02 | -.01 |  |  |
|  |  |  |  |  |  |  |  |  |  |  |  |  |  |  |
| 11. IC_30 | 1,752 | 4.59 | 9.40 | .06** | .02 | -.03 | -.03 | -.12** | -.00 | .03 | .06* | .03 | .11** |  |
|  |  |  |  |  |  |  |  |  |  |  |  |  |  |  |
| 12. IC_70 | 1,750 | 3.68 | 6.91 | -.05* | -.13** | -.09** | .05 | .15** | .03 | .17** | .08** | -.02 | -.07** | .12** |
|  |  |  |  |  |  |  |  |  |  |  |  |  |  |  |

*Note.* *M* and *SD* represent mean and standard deviation, respectively. STER = Stereotypes; PR-DIS = Prejudice and discrimination; ASD = Aging Semantic Differential; ABST = Age-based Stereotype Threat; ACT = Prescriptive age stereotypes – Activation; DIS = Prescriptive age stereotypes – Altruistic Disengagement; ROPE_P = Positive ageism; ROPE_N = Negative ageism; WHO =Ageism Experiences; EVAL = evaluations of younger relative to older adults. Positive values indicate more favourable evaluations of younger adults compared to older adults. IC_30 = number of friends 30 or younger; IC_70 = number of friends 70 or older. For all other variables, higher values indicate greater negativity: more negative stereotypes, stronger prejudice and discrimination, greater age-based stereotype threat, stronger endorsement of activation and altruistic disengagement, higher levels of ageism (as assessed by the ROPE and WHO scales), and more frequent contact with individuals aged 30 or younger / 70 or older. * indicates *p* < .05. ** indicates *p* < .01.

| **Table S5** | | | | |
| --- | --- | --- | --- | --- |
| *WHO-A-TOPS: Multigroup CFA Results. Global Fit Measures for the Exact Measurement Equivalence of the Two-Factor Model, Countries: Czech Republic, Germany, Israel, and the United Kingdom* | | | | |
|  | Chi2(df) | RMSEA | CFI | SRMR |
| configural | 423.212 (148)^***^ | 0.068 | 0.954 | 0.048 |
| Metric | 406.951 (143)^***^ | 0.075 | 0.934 | 0.070 |
| partial metric | 357.399 (139)^***^ | 0.069 | 0.947 | 0.059 |
| partial scalar | 451.528 (154)^***^ | 0.076 | 0.929 | 0.066 |
| *Note.* CFA=confirmatory factor analysis; RMSEA= root-mean-square error of approximation; CFI= comparative fit index; *SRMR= standardized root-mean-square residual. *** p <0.001; ** p <0.01; * p < 0.05* | | | | |
|  |  |  |  |  |

| **Table S6** | | | | |
| --- | --- | --- | --- | --- |
| *WHO-A-TOPS: Multigroup CFA Results. Global Fit Measures for the Exact Measurement Equivalence of the Two-Factor Model, Age Groups: 20-45 years, 46-65 years, and 66+ years* | | | | |
|  | Chi2(df) | RMSEA | CFI | SRMR |
| configural | 257.897 (93)^***^ | 0.063 | 0.958 | 0.044 |
| Metric | 270.032 (109)^***^ | 0.058 | 0.959 | 0.046 |
| Scalar | 306.759 (125)^***^ | 0.056 | 0.955 | 0.049 |
| *Note.* CFA=confirmatory factor analysis; RMSEA= root-mean-square error of approximation; CFI= comparative fit index; *SRMR= standardized root-mean-square residual. *** p <0.001; ** p <0.01; * p < 0.05* | | | | |
|  |  |  |  |  |

**Figures**

**Figure S3**

*Mean Adjusted WHO-A-TOPS for The Two-Factor Solution Across Countries*

**
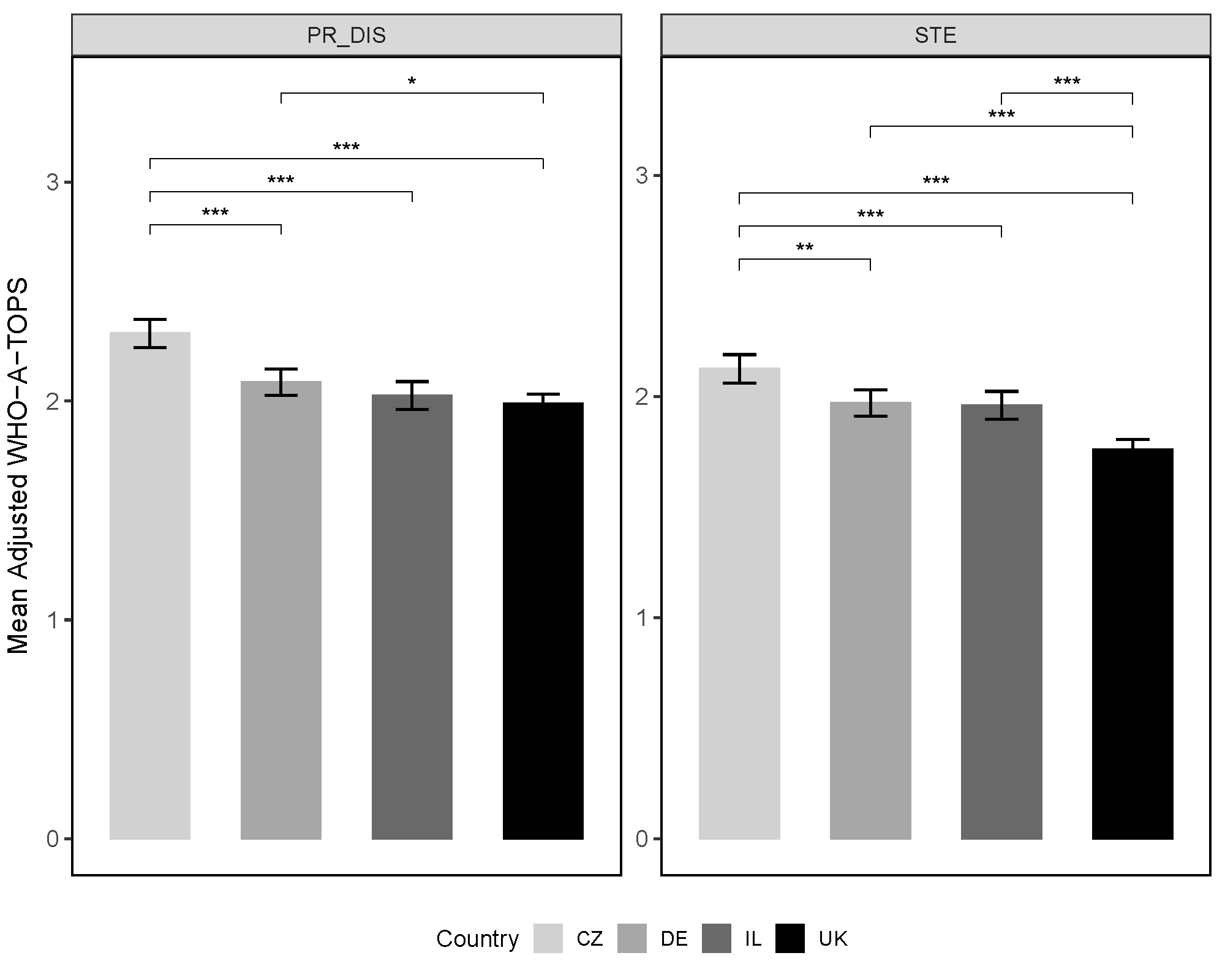
**

*Note.* PR_DIS = Prejudice/Discrimination; STE = Stereotypes; Czech Republic (CZ), Germany (DE), Israel (IL), and the United Kingdom (UK). *indicates *p* < .05, **indicates *p* < .01, *** indicates *p* < .001

**Figure S4**

*Mean Adjusted WHO-A-TOPS for The Two-Factor Solution Across Age Groups*

**
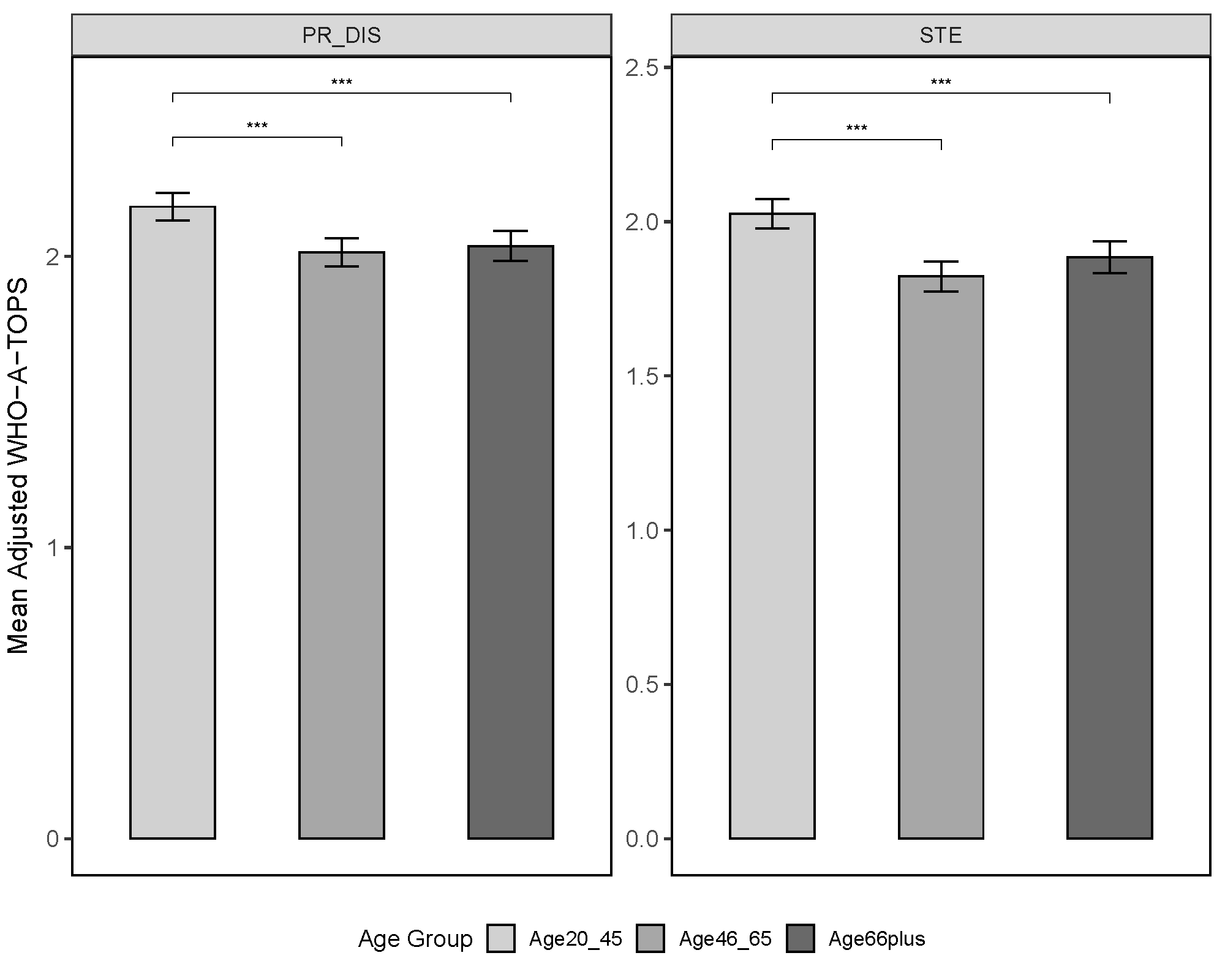
**

*Note.* PR_DIS = Prejudice/Discrimination; STE = Stereotypes; *** indicates *p* < .001

**References**

1. Raykov T. Estimation of composite reliability for congeneric measures. Appl Psychol Meas 1997;21(2):173–184

1. A two-factor solution separating stereotypes from prejudice and discrimination is also viable and shows satisfactory measurement invariance across countries and age groups, although the two factors show substantial overlap (for details regarding the 2-factor solution, see the Supplement). [↑](#footnote-ref-1)
